# The Effect of Sleep Quality on Academic Performance: A Systematic Review and Meta-Analysis Study

**DOI:** 10.3390/bs16050634

**Published:** 2026-04-23

**Authors:** Jing Zhou, Yi Liu, Chunyan Yue, Meng Wang, Keting Chen, Kevin P. Rosales

**Affiliations:** 1 College of Teacher Education, Ningbo University, No. 818 Fenghua Road, Jiangbei District, Ningbo 315211, China; zhoujing@nbu.edu.cn (J.Z.);; 2 Department of Child, Adolescent, and Family Studies, California State University, San Bernardino 5500 University Pkwy, San Bernardino, CA 92407, USA; kevin.rosales@csusb.edu

**Keywords:** sleep quality, academic performance, school-age, meta-analysis

## Abstract

Researchers have long speculated that sleep quality is tied to academic performance. This paper examines this relationship through a meta-analysis using the PRISMA 2020 guidelines. To clarify the relationship between sleep quality and academic performance, this study examined data from 72 independent effect sizes extracted from 59 articles involving 163,357 participants. The results indicate a modest positive correlation between sleep quality and academic performance (*r* = 0.17). Factors such as social jetlag significantly but negatively moderated the relationship between sleep quality and academic performance (*r* = −0.104), while sleep duration showed a significant positive correlation (*r* = 0.132). The school subject (*Q* = 14.986), age of participants (*Q* = 8.606), and culture (*Q* = 4.585) were significant moderators. Furthermore, while the research method did not significantly moderate the relationship between sleep quality and academic performance, the positive and significant association is robust across self-reported, objectively measured, and other-reported sleep quality, despite descriptive differences in effect-size magnitude. Given that sleep is an important physiological process associated with learning and development, students’ sleep quality warrants careful attention.

## 1. Introduction

Sleep influences human physiological functions, affects physical and mental health (Galland et al., 2012; Wong et al., 2013), and plays a crucial role in the development of an individual’s brain and physiological functions (Bell & Zimmerman, 2010). However, there is no unified definition for sleep quality, which is variably measured in terms of factors such as sleep duration, sleep waking, wakefulness, and sleep efficiency (Musshafen et al., 2021; National Sleep Foundation, 2024). Disrupted or insufficient sleep at night can lead to obesity in individuals (Geoffroy et al., 2013), poorer cognitive function (Wickens et al., 2015), and behavioral problems (Lam et al., 2011), and it can increase the likelihood of accidental falls for children (Boto et al., 2012).

Sleep plays a fundamental role in supporting neurocognitive processes that are essential for learning and academic performance. A large body of research indicates that sleep contributes to memory consolidation, attentional regulation, executive functioning, and emotional regulation through interactions among neuroendocrine processes, neural plasticity, and brain network activity (Curcio et al., 2006; Masi et al., 2023). In particular, sleep stages such as slow-wave sleep and rapid eye movement (REM) sleep have been implicated in the consolidation and integration of newly learned information and the stabilization of memory traces (Parrino et al., 2009; Roehrs & Roth, 2000). When sleep is disrupted or insufficient, these neurocognitive processes may be compromised, leading to reduced attentional control, slower information processing, and difficulties with learning and academic engagement (Baglioni et al., 2016; Wickens et al., 2015). Collectively, this literature suggests that sleep quality may influence academic performance through its effects on multiple cognitive systems that support learning.

Sleep also influences academic performance. For example, sleep quality significantly predicts adolescents’ final grades: individuals with higher sleep quality have a lower risk for poor academic performance (Adelantado-Renau et al., 2019a; Tonetti et al., 2015). The strength of the relationship between sleep quality and academic performance also varies. Correlation coefficients between sleep quality and academic performance range from weak (Armand et al., 2021; Faught et al., 2017b; Singh et al., 2018) to moderate (Tonetti et al., 2015) and strong (Lien et al., 2020). Some moderating variables, such as age, learning subject, and culture, influence the strength of this relationship. For example, age is a crucial moderator. Studies show that the daily sleep of upper-grade primary school children has a cumulative positive effect on academic performance (Fonseca & Genzel, 2020). However, the correlation strength decreases among middle school students (Lien et al., 2020). Studies with preschool children indicate a weak correlation between sleep quality and academic performance (Hoyniak et al., 2020).

Secondly, the school subject affects the relationship between sleep quality and academic performance. Research shows a significant correlation between sleep quality and performance in mathematics, languages, and science (Dubuc et al., 2019). However, the correlation coefficients vary significantly for specific subjects. Some scholars point out that the correlation between sleep quality and language performance is the strongest (Howie et al., 2020). Others indicate a correlation between sleep quality and mathematics performance, but the correlation coefficients vary widely, from null (Singh et al., 2018; Perez-Lloret et al., 2013) to moderate (Zhao, 2022). Additionally, some researchers state that sleep quality significantly predicts performance across various subjects, but the prediction strength does not vary substantially between subjects (Adelantado-Renau et al., 2019a). Although the impact of sleep quality on academic performance has been extensively studied, the impact of sleep quality on academic performance due to differences in learning subject remains an unanswered question.

Lastly, culture might be a potential influencing factor between sleep quality and academic performance. Intercultural and multicultural perspectives are essential for understanding how children’s learning and development relate to and are shaped by their diverse contexts (Begeny et al., 2021). Cross-national differences in school time provide important context for understanding cultural variation in sleep and academic outcomes. According to an OECD report, students in China spend substantially more total time on learning activities than students in most OECD countries. For example, PISA 2018 data show that students in regions of China (Beijing–Shanghai–Jiangsu–Zhejiang) reported approximately 54 h per week spent on learning activities, including time at school and after school, compared with an OECD average of about 44 h per week (OECD, 2019). In contrast, official OECD indicators show that students in many Western countries, including the United States, European countries, and Australia, receive an average of 805 h of compulsory instruction per year in primary school and 916 h per year in lower secondary school, corresponding to roughly 4–5 h of instruction per day, with school days typically ending by mid-afternoon (OECD, 2023). These systemic differences in educational time demands may contribute to cross-cultural variation in sleep duration and sleep quality among children and adolescents. One study also indicated that Chinese children sleep nearly an hour less on average than American children (Touchette et al., 2007). Surveys show that approximately 94.4% of Chinese children experience sleep insufficiency, with homework duration being the primary factor affecting sleep (Tao et al., 2019). Concerning sleep quality, the rate of sleep disorders combined with reports in foreign countries is 20–25%, much lower than the 35.1% figure for Chinese children (Tang, 2020). Whether there are significant cultural differences in how sleep quality affects students’ academic performance has yet to be empirically studied.

Although research consistently shows that sleep quality influences academic outcomes, the magnitude of this relationship varies considerably across studies (Armand et al., 2021; Lien et al., 2020; Tonetti et al., 2015). These inconsistencies may arise from moderating variables such as age, cultural background, specific components of sleep (e.g., duration, latency, social jetlag, bedtime), and methodological factors (e.g., subjective versus objective assessment of sleep and academic performance). For example, age-related developmental differences in sleep structure may alter how sleep quality relates to learning across early childhood and adolescence (Fonseca & Genzel, 2020; Lien et al., 2020; Hoyniak et al., 2020), while cultural factors may influence both sleep behaviors and academic demands (Tang, 2020; Tao et al., 2019). Likewise, studies using self-reported sleep measures often yield stronger associations with academic outcomes than those using more objective methods, suggesting that methodological variability may contribute to the heterogeneity in findings (Adelantado-Renau et al., 2019a). Clarifying how these moderators shape the sleep–academic performance link is therefore essential for understanding when and for whom sleep quality most strongly predicts academic success.

While prior research has examined the relationship between sleep and academic outcomes, existing syntheses have often focused on sleep duration or general sleep habits rather than the broader construct of sleep quality. Moreover, many prior studies have examined specific developmental stages or limited sets of moderators, leaving uncertainty regarding how factors such as cultural context, developmental stage, and specific sleep components may shape the strength of the sleep–achievement relationship. The present meta-analysis extends prior work by synthesizing evidence across childhood and adolescence while simultaneously examining multiple theoretically relevant moderators, including sleep components, demographic characteristics, cultural context, and measurement methods. By integrating these moderators within a unified framework, this study aims to provide a more comprehensive understanding of when and for whom sleep quality is most strongly associated with academic performance.

Because there is a great deal of research on how sleep affects academic performance, different studies often focus on different moderating variables. This makes it more difficult to clarify the relationship between sleep, academic performance, and those moderators. The gaps within the literature lead to further exploration of how sleep quality relates to academic performance and what factors influence this relationship. This meta-analysis study is informed by the biopsychosocial model (Engel, 1977) and ecological systems theory (Bronfenbrenner, 1979), which together highlight the multifaceted nature of academic performance as influenced by individual biological states (e.g., sleep quality and duration, age), psychological processes (e.g., daytime dysfunction, social jetlag), and contextual factors (e.g., cultural experiences). Thus, the current study aims to employ a meta-analytical approach to focus on the following research questions: 1. What is the strength of the relationship between sleep quality and academic performance? 2. How do moderating variables affect the relationship between sleep quality and academic performance?

## 2. Method

This systematic review and meta-analysis was conducted largely in accordance with the Preferred Reporting Items for Systematic Reviews and Meta-Analyses (PRISMA) 2020 guidelines. The PRISMA checklist has been completed and is provided in the Supplementary Materials. The review was not registered in a public registry. We confirm that the dataset compiled for this meta-analysis has not been previously published or used in prior manuscripts, conference proceedings, or dissertations by the authors or others. All analyses presented in this paper are original to the present study.

### 2.1. Information Source and Search Strategy

English and Chinese literature were included in this study due to the language of the author(s). We first searched Chinese sources using native-language keywords to map core terminology, then translated and cross-validated terms against English abstracts of Chinese papers and definitions in English-language studies. Chinese literature searches mainly used databases such as CNKI (China National Knowledge Infrastructure), and Wanfang Database. The keywords are consistent with the English database search. Additionally, a backward search was performed based on the citations of the retrieved literature to find related studies. English literature searches were conducted in Ebscohost, Taylor & Francis, Wiley, Elsevier Science Direct, Web of Science, Springer Link, and Theses Full Text Database. In order to conduct an exhaustive search through our query of online databases, we included the following search terms, making sure to select for articles that included both the word sleep, or a related synonym, and the word academic achievement (denoted by the connecting AND): “sleep quality”, “sleep problem”, “sleep duration”, “bedtime”, “sleep latency”, “daytime dysfunction” OR “social jetlag” AND “academic success”, “academic performances”, “GPA”, “maths”, “science”, “STEAM” OR “language” in English, and “睡眠质量”, “睡眠时间”, “睡眠节律”, OR “睡眠效率” AND “学业成绩”, “学业表现”, “成绩”, “数学”, “科学”, OR “语文” in Chinese.

Our decision to focus on studies published from August 2013 to April 2024 was driven by empirical trends in the literature rather than assumptions about the irrelevance of earlier work. As illustrated by publication trends reviewed during study planning, research on children’s sleep problems and their academic or cognitive correlates increased markedly beginning around 2014. This rise coincided with heightened public health and educational attention to sleep issues among children, particularly in China, where prevalence reports peaked around the time this project was initiated (2023). To capture this rapid growth while maintaining a manageable and conceptually coherent scope, we initially targeted the most recent decade of research and extended the search window back to 2013 to ensure coverage of the emerging literature. To assess whether this pattern was region-specific, we also examined publication trends in international databases (e.g., Web of Science) and observed a similar increase in sleep–academic performance studies during the same period. Based on this convergence of domestic and international research trajectories, we limited the search period to 2013–2023 to focus on contemporary evidence that reflects current educational systems, sleep measurement practices, and analytic approaches; however, as the project timeline extended, we updated the search to include studies published in 2024, resulting in a final window of 2013–2024 at the time of manuscript submission.

Following the PICO framework, this review included: (P) typically developing children and adolescents aged 0–18 years; (I/E) sleep quality assessed via self-report, informant report, or objective measures; (C) subgroup comparisons across age, culture, or measurement method; and (O) academic performance outcomes, including grades and standardized academic assessments.

### 2.2. Eligibility Criteria

Individual studies were selected according to the following criteria: (1) quantitative research, with (a) clear sample size and measurement (i.e., each included study was required to explicitly report the sample size and provide sufficient methodological detail about the measures used) and (b) presentation of required initial effect values (i.e., r-values, F-values, d-values, odds ratio); (2) study subjects were reported as typical developing children aged 0–18 years; (3) research data had not been previously published (if reused, literature with the most independent effect values was chosen); and (4) literature was written in Chinese or English in full-text. Studies that did not meet any of the inclusion criteria outlined above were excluded. Such studies include any purely qualitative studies, observational study designs, and literature reviews without extractable effect sizes; studies focusing on subjects aged 18 years or older; studies with subjects reported as atypical developing children; or, cases in which the full text was not available (i.e., conference proceedings with abstract only) or not written in English or Chinese.

**Figure 1:**
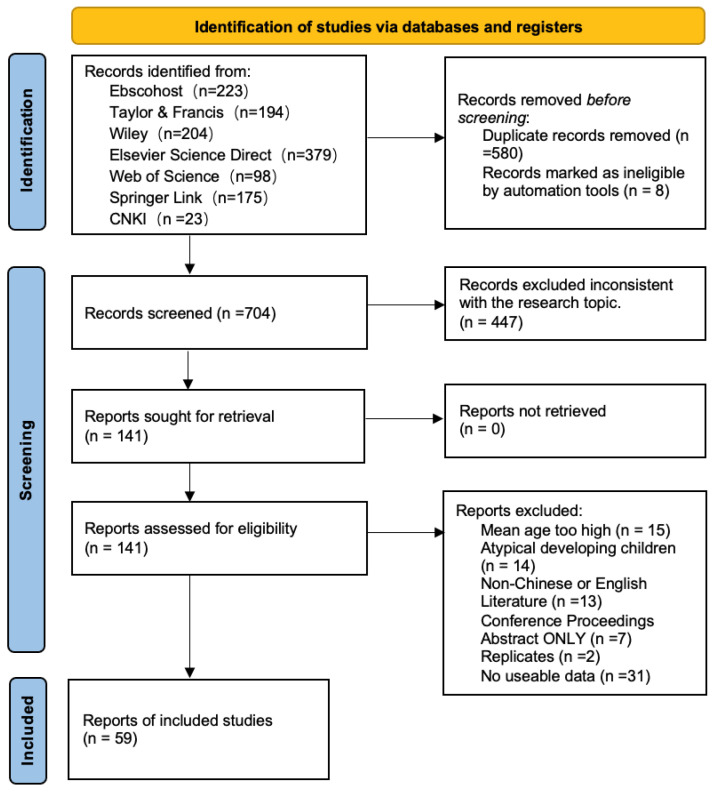
PRISMA 2020 flowchart of literature searches and study selections.

We did not formally assess journal quality using ranking systems (e.g., impact factor thresholds) as an inclusion criterion. However, the majority of included studies were published in established peer-reviewed journals indexed in major academic databases (e.g., Web of Science, Scopus, CNKI). During the screening process, we carefully reviewed journal information to ensure that outlets met standard scholarly publishing practices and were not identified as predatory sources. In addition, all included studies—regardless of publication type (including unpublished theses and dissertations)—were required to meet our methodological inclusion criteria (e.g., clear sample description, valid measurement tools, and reportable effect sizes). Finally, 59 articles were selected, including 55 in English and 4 in Chinese, accounting for 72 independent effect values. The process of screening and selecting studies is illustrated in Figure 1.

### 2.3. Coding

The necessary coding features included literature information (authors, publication year), sample size, age of participants, measurement (i.e., actigraphy, self-report and other-report), and cultural background (i.e., Eastern culture and Western culture) (see Table 1).

For the age variable, three analyzable groups were defined aligned with the Chinese education system: 0–6 years, 7–12 years, and 13–18 years, corresponding to preschool age, primary school age and secondary school age, respectively. If a study sample spanned multiple educational stages, we used the age range reported in the literature. When age-specific effect sizes by educational stage were not available (e.g., ages 5–10, including both preschool and primary school age), the study was coded as “other”. For the cultural variable, Western culture refers to countries primarily located in Europe and other regions historically influenced by European cultural traditions, including the United States, Canada, Australia, and New Zealand. Eastern culture refers primarily to countries located in Asia, including East, Southeast, and parts of Western Asia, including China, Japan, Korea and India (van de Vijver, 2013; Vignoles et al., 2016). Research design and measurement characteristics that were included as moderators in the analyses were as follows: (1) measurement (i.e., the measurement of sleep was categorized into actigraphy, self-report, and report by others; the measurement of academic performance was sorted by school transcript, self-report and report by others), and (2) other moderators, including bedtime, social jetlag, sleep latency, and sleep duration (i.e., above-recommended and below-recommended), and weekend sleep. Finally, the sources of the included data and the publication types were identified (i.e., peer-reviewed journal articles vs. dissertations/theses as grey literature) and reported in Table 1.

Additionally, because the concept of sleep quality is operationalized differently across studies, clear inclusion rules were applied when extracting effect sizes. For the primary meta-analysis, sleep quality was defined broadly to include composite indices or overall indicators of sleep functioning reported in the original studies. These measures included validated multi-item scales (e.g., the Pittsburgh Sleep Quality Index [PSQI]), global sleep quality scores, and objective indicators reflecting overall sleep efficiency obtained through actigraphy or similar devices. When studies reported multiple sleep indicators, a single composite or global index of sleep quality was prioritized when available.

In contrast, analyses of specific sleep components were conducted separately. When studies reported individual sleep parameters—such as sleep duration, bedtime, sleep latency, or social jetlag—these indicators were extracted and analyzed in moderator analyses examining whether specific aspects of sleep showed differential associations with academic performance. Consequently, the number of effect sizes differs across these component-level analyses (e.g., sleep duration n = 55; bedtime n = 8), reflecting the availability of these specific variables within the included studies.

In this study, only one member conducted the article search, but the team leader reviewed the results. Two members were trained for coding and coded all studies independently. The interrater reliability, indicated by Cohen’s kappa, was 0.93, indicating that the literature coding in this study was relatively effective and accurate. The two coders discussed discrepancies in coding, and a consensus was reached.

### 2.4. Risk-of-Bias Assessment

To evaluate the methodological quality of the included studies, a risk-of-bias assessment was conducted for each study. Because most studies included in the meta-analysis used observational or correlational designs, we applied criteria adapted from established quality assessment frameworks commonly used in meta-analytic research (e.g., Higgins & Green, 2011; Sterne et al., 2016). Each study was evaluated across several domains relevant to observational research, including:

(1) Clarity of sample description and recruitment procedures;
(2) Reliability and validity of sleep measurement;
(3) Reliability and validity of academic performance measurement;
(4) Adequacy of statistical reporting for effect size extraction;
(5) Potential confounding variables reported or controlled in the analysis.

### 2.5. Calculation and Analysis of Effect Sizes

Pearson’s r, the correlation coefficient between the sleep and school performance variables, served as the effect size estimation. If r could not be obtained from the publication, other given statistics (e.g., *p*, χ^2^, or F) were used to estimate r. The conversion is calculated as follows (Harrer et al., 2021):

$$r=\beta \times 0.98+0.05\lambda \;(-0.5<\beta <0,\;\lambda =-1;\;0<\beta <0.5,\;\lambda =1);\;r=\sqrt{\frac{d^{2}}{d^{2}+4}}=\sqrt[{}]{\frac{t^{2}}{t^{2}+df}}=\ln\left(OR\right)\frac{\sqrt{3}}{\pi }=\sqrt{\eta^{2}}.$$

Because r has undesirable statistical properties, correlations were transformed to Fisher’s z values. Weighted overall effect sizes and confidence intervals were calculated. For ease of interpretation, overall effect sizes were converted back into r. If two or more assessments of the same sleep variable were reported separately, average effect sizes were calculated. If studies assessed school performance by measuring participants’ math, reading, or language ability, the average of the reported outcome scores was used as a school performance indicator. If grades were written for different disciplines separately, their average was used as the school performance measurement. Finally, 72 independent effect sizes with 163,357 participants were obtained based on the above approach.

### 2.6. Data Analysis

The current study utilized Comprehensive Meta-Analysis (CMA) 3.0 for statistical analysis. Firstly, heterogeneity was assessed using Cochran’s Q-test and I^2^ statistic, and based on these results, either a fixed-effect model or a random-effects model was chosen to compute the effect sizes. An I^2^ value exceeding 70% indicates a significant heterogeneity among effect sizes. Secondly, publication bias was examined by funnel plots, Rosenthal’s failsafe N, and Egger’s test (Egger et al., 1997). Finally, the main effect and moderating effects were analyzed with the selected model. The moderating variables explored in this study included age, cultural background, sleep assessment methods, sleep components (social jetlag, sleep efficiency, sleep latency, sleep duration, bedtime), and school subjects. It is important to note that subgroup analysis for moderating effects was only conducted when each subgroup contained four or more independent effect sizes (Collier et al., 2016).

## 3. Results

### 3.1. Risk-of-Bias Results

Overall, most studies demonstrated moderate to low risk of bias across the evaluated domains. The majority of studies clearly reported sample characteristics and provided sufficient statistical information for effect size extraction. Variability was most evident in the measurement of sleep quality, as studies used a range of subjective and objective indicators. However, these differences reflect typical methodological variation in sleep research rather than systematic bias.

### 3.2. Publication Bias

**Figure 2:**
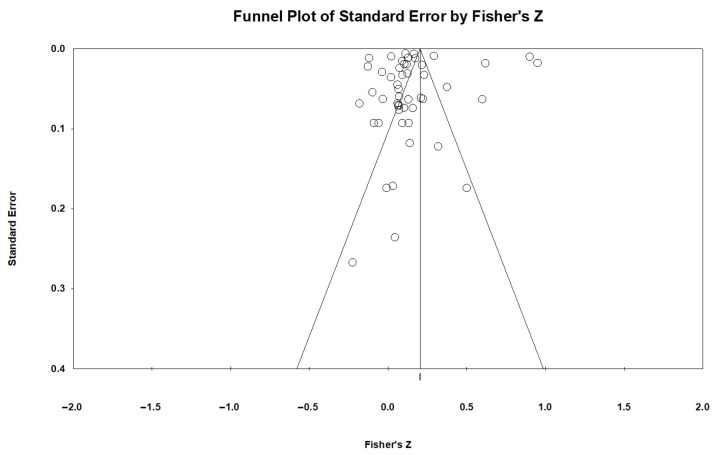
Funnel plot based on Fisher’s Z scores.

The results of the publication bias assessment are shown in Figure 2. The funnel plot indicates that most independent effect sizes are concentrated above the graph and cluster around the overall effect size, displaying a nearly symmetrical trend. The fail-safe N is 8208, significantly exceeding the standard of 5k + 10 (in this study, k = 72). The results of Egger’s linear regression indicate the standard errors of correlations did not significantly predict correlations among studies (*t* = 0.94, *p* = 0.16). Thus, the likelihood of overturning the meta-analysis conclusion is low.

### 3.3. Heterogeneity Testing

**Figure 3:**
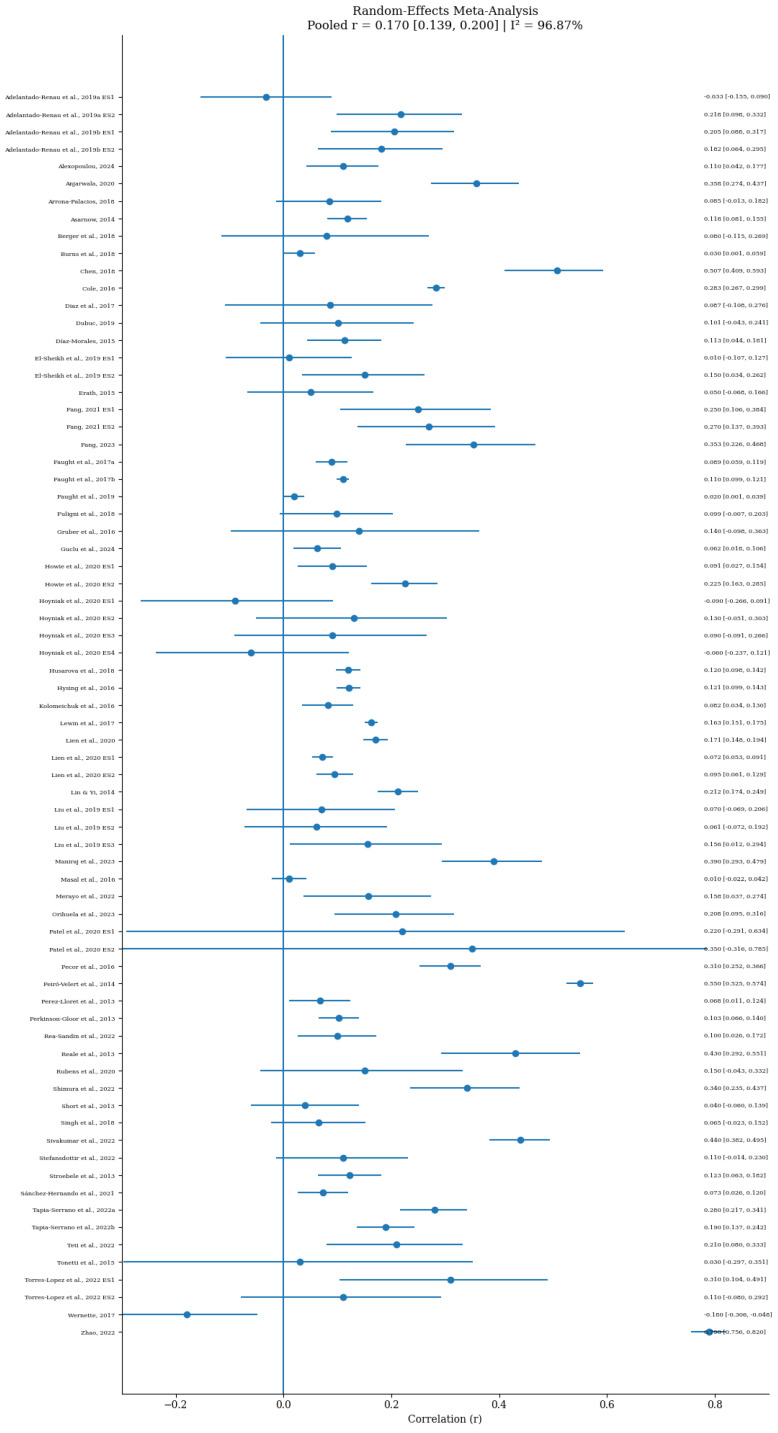
Forest plot. The studies included are (Adelantado-Renau et al., 2019a, 2019b; Alexopoulou et al., 2024; Anjarwala et al., 2020; Arrona-Palacios & Díaz-Morales, 2018; Asarnow et al., 2014; Berger et al., 2018; Burns et al., 2018; Chen, 2018; Cole, 2016; Diaz et al., 2017; Díaz-Morales et al., 2015; Dubuc et al., 2019; El-Sheikh et al., 2019; Erath et al., 2015; Fang, 2021; Fang et al., 2023; Faught et al., 2017a, 2017b, 2019; Fuligni et al., 2018; Gruber et al., 2016; Guclu et al., 2024; Howie et al., 2020; Hoyniak et al., 2020; Husarova et al., 2018; Hysing et al., 2016; Kolomeichuk et al., 2016; Lewin et al., 2017; Lien et al., 2020; Lin & Yi, 2014; J. Liu et al., 2019; Maniraj et al., 2023; Masal et al., 2016; Merayo et al., 2022; Orihuela et al., 2023; Patel et al., 2020; Pecor et al., 2016; Peiró-Velert et al., 2014; Perez-Lloret et al., 2013; Perkinson-Gloor et al., 2013; Rea-Sandin et al., 2022; Reale et al., 2013; Rubens et al., 2020; Sánchez-Hernando et al., 2021; Shimura et al., 2022; Short et al., 2013; Singh et al., 2018; Sivakumar et al., 2022; Stefansdottir et al., 2022; Stroebele et al., 2013; Tapia-Serrano et al., 2022a, 2022b; Teti et al., 2022; Tonetti et al., 2015; Torres-Lopez et al., 2022; Wernette & Emory, 2017; Zhao, 2022)^1^.

The results of heterogeneity testing are shown in Figure 3. The forest plot reveals that while some segments parallel to the x-axis are relative short with significant overlap, most segments exhibit substantial length variations and significant differences in overlap. This indicates that the effect sizes across studies are different, which preliminarily shows that the literature samples included in this study exhibit high heterogeneity. 

Further heterogeneity analysis was conducted with Q and I^2^ tests. A significant heterogeneity was found among the effect sizes, *Q* = 2267.18, *p* < 0.001, with an I^2^ value of 96.87%, suggesting the use of a random-effects model for effect size estimation. Furthermore, this study exhibited significantly high heterogeneity among effect sizes with a T^2^ = 0.020, indicating that the variations among effect sizes were influenced by certain moderating variables, warranting a secondary meta-analysis.

Sensitivity analysis was conducted using the one-study-removed method. The result demonstrates that the coefficients remained stable after excluding any single study in both the fixed-effects model (0.142~0.145) and random-effects model (0.165~0.173). All 95% CI exclude 0 (*p* < 0.001), indicating the robustness of the study’s conclusions.

### 3.4. The Main Effect Between Sleep Quality and Academic Performance

Sleep quality and academic performance were found to be significantly and positively correlated, *r* = 0.17, *p* < 0.001, 95%CI = [0.139, 0.200], with a low to moderate strength according to the effect size criteria (Gignac & Szodorai, 2016). Sleep quality accounts for nearly 3% of the variance in academic performance. The prediction interval (PI) analysis showed a high heterogeneity among future studies with a 95%CI [−0.0017, 0.411], *t* = 0.121, indicating a similar potential distribution range of effect size in future studies. 

### 3.5. The Impact of Moderating Effects

This study conducted four types of meta-analyses to analyze the moderating effects on the strength of the relationship between sleep quality and academic performance, including (1) the different components of sleep; (2) the different school subjects; (3) the different demographic information; and (4) the different measurement methods for academic performance. A summary of the meta-analytic results is reported in Table 2.

**Various Components of Sleep.** The moderating effects of varying sleep quality indicators on the relationship between sleep quality and academic performance were significant, *Q* = 108.694, *p* < 0.001. The average effect size for bedtime was negatively but nonsignificantly correlated with academic performance, *r* = −0.24, *p* = 0.054, 95%CI = [−0.457, 0.004]. Social jetlag was significantly and negatively correlated with academic performance, *r* = −0.104, *p* < 0.001, 95%CI = [−0.138, −0.070], suggesting that a more extensive social jetlag is associated with poorer academic performance. Latency was also negatively correlated with academic performance, *r* = −0.154, *p* < 0.001, 95%CI = [−0.214, −0.092]. Daytime dysfunction was significantly but negatively associated with academic performance, *r* = −0.238, *p* = 0.006, 95%CI = [−0.394, −0.007]. Sleep duration was positively and significantly correlated with academic performance *r* = 0.132, *p* < 0.001, 95%CI = [0.099, 0.164].

**Different School Subjects.** The moderating effect of school subjects on the relationship between the sleep quality and academic performance is significant, *Q* = 14.986, *p* = 0.001. Sleep quality is positively and significantly correlated with mathematics performance, *r* = 0.206, *p* < 0.001, 95%CI = [0.109, 0.298], and language performance, *r* = 0.213, *p* < 0.001, 95%CI = [0.116, 0.305], but not with science performance, *r* = 0.020, *p* = 0.840, 95%CI = [−0.176, 0.215]. There is a low positive correlation between sleep quality and academic performance in math and languages, indicating that sleep quality is a small but non-negligible influencing factor for academic performance, and this influence varies significantly across different subjects.

**Different Demographic Information.** The age of the samples in individual studies was analyzed with three categories: 0–6 years old (early childhood/pre-school), 7–12 years old (school-aged/elementary), and 13–18 years old (school-aged/secondary school). The moderating effect of age on the relationship between sleep quality and academic performance is significant, *Q* = 8.606, *p* = 0.014. The correlations between sleep quality and academic performance are positive but nonsignificant for early childhood children aged 0–6 years, *r* = 0.072, *p* = 0.096, 95%CI = [−0.013, 0.157], but positive and significant for 7–12 years old school-aged children, *r* = 0.230, *p* < 0.001, 95%CI = [0.161, 0.296], and for 13–18 years old school-aged adolescents, *r* = 0.139, *p* < 0.001, 95%CI = [0.093, 0.185].

The moderating effect of culture on the relationship between sleep quality and academic performance is significant, *Q* = 4.585, *p* = 0.032. The correlation between sleep quality and academic performance is positive and significant among children from Eastern culture, *r* = 0.284, *p* < 0.001, 95%CI = [0.154, 0.404], and among those with Western cultural backgrounds, *r* = 0.138, *p* < 0.001, 95%CI = [0.108, 0.168].

**Different Research Methods.** Research methods nonsignificantly moderate the relationship between sleep quality and academic performance, *Q* = 3.442, *p* = 0.179. The average effect size was 0.16 (*p* < 0.001, 95%CI = [0.123, 0.195]) for self-reported sleep quality and academic performance, 0.127 (*p* = 0.003, 95%CI = [0.044, 0.209]) for sleep quality measured by actigraphy, and 0.284 (*p* < 0.001, 95%CI = [0.139, 0.417]) for sleep quality reported by others. Similarly, the moderating effect of different academic performance measurement methods was not significant (*Q* = 4.03, *p* = 0.133). The average effect size between sleep quality and self-reported academic performance was 0.127 (*p* < 0.001, 95%CI = [0.084, 0.170]), between sleep quality and school report cards was 0.193 (*p* < 0.001, 95%CI = [0.139, 0.245]), and between sleep quality and academic performance reported by others was 0.121 (*p* = 0.002, 95%CI = [0.043, 0.197]).

## 4. Discussion

### 4.1. The Relationship Between Sleep Quality and Academic Performance

Due to the lack of consistency in the correlations between sleep quality and academic performance from existing studies, the current study employed a meta-analytic approach that revealed a positive correlation between sleep quality and academic performance. Although sleep quality only accounted for a modest amount of variance in academic performance, it is nonetheless an influential factor that cannot be overlooked. Poor sleep quality can lead to suboptimal educational outcomes (Taras & Potts-Datema, 2005; Musshafen et al., 2021). Researchers widely believe that sleep is crucial in regulating an individual’s physiological and psychological well-being, facilitating energy, physical recovery, and emotional regulation (Thomas et al., 2020). Poor sleep quality can lead to headaches (Luntamo et al., 2012), persistent fatigue (Rimes et al., 2007), and physical discomfort, affecting a student’s comprehension and mastery of the learning materials. All of these negative physiological outcomes can directly impact academic performance in the classroom.

Although the present meta-analysis demonstrates a significant association between sleep quality and academic performance, it is important to recognize that sleep quality may not represent a singular causal mechanism. Rather, sleep quality likely reflects the influence of broader psychological and contextual factors that also shape academic functioning. For example, mental health conditions such as anxiety and depression are known to disrupt sleep patterns while simultaneously impairing concentration, motivation, emotional regulation, and executive functioning—processes that are directly relevant to academic performance. In such cases, sleep disturbances may serve as both a consequence of psychological distress and an indirect pathway through which broader mental health challenges manifest in academic difficulties.

Furthermore, sleep quality, academic performance, and their relationship can also be influenced by factors such as individual lifestyle, family socioeconomic status, and sleep environment quality, which have yet to be adequately explored in previous research. These research findings suggest that in addition to examining the direct impact of sleep on academic performance, investigating mediating variables through which sleep affects academic performance, thus assessing the explanatory power of sleep on academic performance may be needed. Moreover, existing studies on the relationship between sleep quality and academic performance mainly employ cross-sectional designs, treating sleep quality and academic performance as stable variables rather than investigating how changes in sleep quality relate to changes in academic performance. These factors might contribute to the weak relationship between sleep quality and academic performance.

### 4.2. The Moderating Effects on the Relationship Between Sleep Quality and Academic Performance

**Social Jetlag.** Social jetlag is commonly defined as the absolute difference between the midpoint of sleep on workdays (or school days) and the midpoint of sleep on free days (e.g., weekends) (Wittmann et al., 2006). Consistent with previous research (Hysing et al., 2016), this meta-analysis study found a negative but significant effect size of social jetlag on academic performance. A higher level of social jetlag reflects a greater discrepancy between weekday and weekend sleep timing, which has been associated in prior research with patterns of weekday sleep restriction and longer sleep duration on weekends (Chang & Jang, 2019; Y. Liu et al., 2022). Prolonged social jetlag implies that individuals spend an extended period in a state of sleep deprivation, which can adversely affect their emotions, cognition, and behavior (Lin & Yi, 2014; Pasch et al., 2010). Additionally, this study found that the positive and significant average effect size of weekend sleep duration on academic performance, corroborating the relationship between social jetlag and academic performance. Sufficient sleep on weekends compensates for the sleep deficit during weekdays, making it easier for individuals to cope with new learning tasks during the week.

**Sleep Duration.** Meta-analysis results reveal that sleep duration positively and significantly moderates the relationships between sleep quality and academic performance. Sufficient sleep provides the necessary nocturnal brain activity for the neurocognitive system. It aids individuals in handling complex tasks requiring various higher-order cognitive functions, ultimately contributing to better academic performance (Smith et al., 2020). However, this does not imply that longer sleep duration is always better. Meta-analysis results also indicate that insufficient and excessive sleep can negatively impact an individual’s academic performance, in line with previous research (Ünalan et al., 2013). The possible explanation is that sleep deprivation affects an individual’s cognitive processing and indirectly exacerbates the negative impact of daytime sleepiness on academic performance (Fuligni et al., 2018). Excessive sleep also negatively affects academic performance, with existing research indicating increased risks of low academic performance. Some scholars, however, argue that individuals with excessively long sleep may not need to reduce their sleep duration and that unstable circadian rhythms and excessive deviations from the optimal sleep duration are more related to lower cognition (Hua et al., 2020).

**Sleep Latency.** Sleep latency, which measures the difficulty of falling asleep, is considered an indicator of sleep quality. Longer sleep latency may signify difficulty falling asleep or even insomnia. Contrary to previous research findings (e.g., Cooper et al., 2012), this study has demonstrated a negative but nonsignificant correlation between sleep latency and academic performance. Although nonsignificant, chronic sleep onset difficulties might lead to sleep deprivation and daytime fatigue, consequently affecting academic performance. Individual sleep onset latency is influenced by various factors, including anxiety, depression, stress, maladaptation to sleep environment, circadian rhythm disruption, and even the emotional state before bedtime (Hajime, 2013). Longer required sleep onset time may also increase an individual’s anxiety about sleep, leading to a vicious cycle that affects overall sleep, anxiety, and, in turn, academic performance. In other words, individuals with insomnia may become anxious about their sleep, which then results in the need for more time to fall asleep, reinforcing their perceptions of sleep difficulties.

**Bedtime.** The average effect size of bedtime on academic performance is negative but also nonsignificant, in contrast to prior research findings (Gruber et al., 2016). Two main reasons can help us understand this result: firstly, bedtime is negatively correlated with sleep quality and cognitive function (Asarnow et al., 2014), and sleep quality and cognitive function are linked to academic performance. Secondly, due to school-imposed restrictions on students’ arrival times, the time students wake up in the morning remains relatively fixed. Going to bed later implies a shorter overall sleep duration, which is why previous research has suggested delaying school start times to reduce the negative impact of sleep deprivation on students (Alfonsi et al., 2020).

Nonetheless, it is important to note that this does not mean that the earlier the bedtime, the better. Some studies suggest a nonlinear relationship between bedtime and academic performance. For most individuals, the optimal bedtime may fall between 22:00 and 23:00 (Hysing et al., 2016). Individuals with later bedtimes might be influenced by their sleep patterns. Sleep patterns can be categorized into morning-type, intermediate-type, and evening-type. Morning-type individuals sleep and wake up early, while evening-type individuals sleep and wake up late, with intermediate-type individuals falling in between (Enright & Refinetti, 2017). Evening-type individuals often have higher energy levels and alertness at night. Therefore, a late bedtime might not necessarily hurt their academic performance compared to the other two types. No studies have explored the relationship between bedtime and academic performance among individuals with different sleep patterns, which could be a direction for future research.

**Age*.*** The meta-analysis results indicate a significant moderating effect of age on the relationship between sleep quality and academic performance. Compared to preschool children aged 0–6, school-aged children show a stronger correlation between sleep quality and academic performance. Specifically, there is no significant correlation between sleep quality and academic performance for children aged 0–6 (*p* > 0.05). This may be due to preschool children transitioning from daytime to nighttime sleep patterns (Zhou & Zhu, 2021), with their overall sleep consisting of both nighttime and daytime sleep. Insufficient nighttime sleep duration and quality in young children can be compensated for by longer naps, meaning that sleep quality has a less significant impact on academic performance. This result also underscores the importance of considering daytime naps in addition to nighttime sleep quality when assessing the influence of sleep on the academic performance of preschool children. Previous research has shown that students across different educational levels commonly experience sleep deprivation and that sleep quality is associated with academic performance in both adolescents (Musshafen et al., 2021) and university students (Bagheri et al., 2025; Nakie et al., 2024), as well as across broader student populations (Curcio et al., 2006). Although the present study did not directly use educational level directly as a moderator, age served as a closely related indicator. Our results indicate that age significantly moderates the association between sleep quality and academic performance. Specifically, a significant association was observed among school-aged children, whereas the association was not statistically significant among preschool-aged children, which aligns with previous studies (e.g., Fonseca & Genzel, 2020; Hoyniak et al., 2020).

The meta-analysis reveals that the correlation between sleep quality and academic performance is strongest in children aged 7–12. As individuals grow, the overall relationship between these two variables exhibits a declining trend, with primary school students showing a slightly stronger association than middle school students. This finding is consistent with previous research (Luntamo et al., 2012; Sadeh et al., 2002). Two potential explanations for this trend are proposed. Firstly, with increasing age, individuals become less sensitive to the effects of sleep deprivation or prolonged wakefulness (Jenni et al., 2005; Taylor et al., 2005). It suggests that individuals may become accustomed to these conditions and may overlook their impact on their own learning. Secondly, this trend is linked to the characteristics of the prefrontal cortex’s development in individuals. Neuroscientific research indicates that early adolescence is a critical period for rapid prefrontal cortex development. During this phase, the prefrontal cortex rapidly evolves in terms of both structure and function, with the development rate gradually decreasing throughout adolescence (Casey et al., 2005). Consequently, inadequate sleep and poor sleep quality during early adolescence exert a greater influence on the prefrontal cortex and individual cognitive functioning, which subsequently results in a more pronounced negative impact on academic performance. Nevertheless, this declining trend does not imply that the influence of sleep quality on students’ academic performance should be gradually disregarded. In fact, as individuals age, the academic workload and learning challenges increase, implying that students experience a continuous reduction in their sleep duration, along with heightened psychological stress and related issues. These factors may serve as mediating variables in the relationship between sleep quality and academic performance.

Additionally, poor sleep quality is associated with psychological problems in adolescents, such as anxiety and depression (Baglioni et al., 2016). Studies also suggest that sleep deprivation is linked to higher suicidal ideation (Winsler et al., 2014) and suicide attempts in adolescents (Fitzgerald et al., 2011). This may be explained by long-term sleep deprivation, which causes physiological and emotional changes in adolescents or affects their emotional regulation abilities (Baum et al., 2013). Excessive negative emotions can hinder standard cognitive processing, affecting cognitive development. Second, sleep impacts students’ neurocognitive functions, including memory consolidation (Tamminen et al., 2010), attentional integration, and executive functions (Maquet, 2001), affecting their performance in complex tasks.

**Cultural Context.** The moderation analysis indicated that cultural context significantly accounted for variability in the strength of the association between sleep quality and academic performance. Although the association was positive and statistically significant in both groups, it was stronger among children from Eastern cultural contexts *(r* = 0.284) than among those from Western cultural contexts (*r* = 0.138). This moderation effect should be interpreted cautiously. Cultural context likely reflects broader systemic influences—including variations in academic expectations, school structures, and sleep norms—rather than a single explanatory mechanism (Bronfenbrenner, 1979; van de Vijver, 2013). For instance, prior evidence suggests that students in some East Asian educational systems experience higher academic demands and longer study hours compared to many Western contexts (OECD, 2019), which may amplify the observable association between sleep and academic functioning. Cultural emphases on effort and academic achievement may also shape how sleep-related difficulties relate to performance (Markus & Kitayama, 1991). However, because these findings are based on correlational data, they do not establish causal mechanisms.

**Differences in Research Methodology.** Research methods did not significantly moderate the relationship between sleep quality and academic performance, indicating that the association is robust across different measurement approaches. Although descriptive differences in effect sizes were observed—such that self-reported sleep quality showed a slightly stronger association with academic performance than objectively measured sleep quality—these differences did not reach statistical significance. This pattern is consistent with prior research suggesting that subjective sleep measures may capture experiential aspects of sleep quality (e.g., fatigue, daytime sleepiness) that are more proximally related to learning and school functioning than objective indicators alone (Adelantado-Renau et al., 2019a; Krystal & Edinger, 2008). Objective measures such as actigraphy, while valuable for assessing sleep parameters like duration and efficiency, may not fully reflect perceived sleep quality or daytime impairment.

Similarly, academic performance measurement methods did not significantly moderate the sleep–achievement relationship. Although school report cards yielded descriptively larger effect sizes than self-reported or other-reported academic performance, these differences should be interpreted cautiously given the nonsignificant moderation results. Notably, many studies relied on parent-reported sleep measures, and prior work has documented discrepancies between parent and child reports of sleep (Dewald et al., 2010), potentially reflecting parents’ limited awareness of children’s subjective sleep experiences and their downstream effects on learning. Together, these findings underscore the importance of using multi-method approaches that integrate subjective, objective, and informant-based assessments to more comprehensively capture sleep quality and its relationship to academic performance.

These findings can also be interpreted through the lens of Bronfenbrenner’s ecological systems theory (Bronfenbrenner, 1979; Bronfenbrenner & Morris, 2006), which conceptualizes child development as occurring within a set of nested environmental systems that interact dynamically over time. Within this framework, sleep behaviors are shaped not only by biological processes but also by contextual influences such as family routines, school schedules, academic expectations, and broader cultural norms. The present findings are consistent with this perspective. For example, the moderating role of cultural context observed in this meta-analysis suggests that differences in educational demands and daily schedules across societies may influence both sleep behaviors and their relationship with academic performance. Similarly, the age-related differences identified in the analysis may reflect developmental changes in the interaction between children’s biological sleep regulation and environmental demands such as school start times and academic workload. From a bioecological perspective, sleep quality can therefore be understood as one component of a broader developmental system in which biological processes, individual behaviors, and environmental contexts jointly influence academic outcomes.

## 5. Limitations and Future Directions

This study examined the relationship between children’s sleep quality and academic performance, with a focus on the moderating effects of age and culture. While the findings support a positive association, several limitations should be acknowledged. First, this study explored only the correlational relationship between sleep quality and academic performance; therefore, causal inferences cannot be made without evidence from experimental or intervention-based research. Future studies may address this by implementing controlled experiments or intervention programs to examine how improving sleep may directly influence cognitive development.

Second, although previous research has indicated an interaction between gender and age (Dewald et al., 2010), our study could not analyze gender as a moderating variable due to limited reporting of gender-specific results. Yet, emerging evidence suggests notable gender differences in sleep patterns (Mannino et al., 2024) and sleep-related problems (Castelnuovo et al., 2025). Whether these gender-based differences affect the link between sleep and cognitive development remains an open question for future investigation.

Third, while we examined correlations across various sleep components and academic outcomes, some key sleep indicators, such as daytime dysfunction and sleep patterns, were not included due to gaps in the existing literature. Addressing these omissions will require more comprehensive reporting in future primary studies.

Fourth, the present meta-analysis revealed substantial heterogeneity among effect sizes (I^2^ = 96.87%), indicating considerable variability in the magnitude of the association between sleep quality and academic performance across studies. High heterogeneity is common in meta-analyses synthesizing research across diverse developmental stages, measurement approaches, and cultural contexts. In the present case, several factors likely contributed to this variability. First, studies differed in how sleep quality was operationalized, including subjective reports, actigraphy-based measures, and parent or teacher reports. Second, academic performance was assessed using a range of indicators, such as standardized tests, school grades, and self-reported achievement. Third, the included samples spanned a wide developmental range from preschool-aged children to adolescents which likely introduces developmental differences in both sleep patterns and academic expectations. Finally, the studies were conducted across multiple cultural contexts, which may influence both sleep behaviors and academic demands.

Several additional limitations of this study should be noted. First, although this meta-analysis included studies published in both English and Chinese—covering a substantial proportion of the available literature—it did not include studies published in other languages. As a result, relevant findings reported in languages such as Japanese, Korean, German, Spanish, or Arabic may not have been captured, which may limit the full global representation of the evidence. Second, this study focused on research published within the most recent decade. While this restricts historical coverage, it also reflects a deliberate emphasis on contemporary evidence. Children’s learning environments, sleep behaviors, and daily routines have changed substantially over the past 10–15 years, alongside increased public and scientific attention to sleep health, rapid technological development, and widespread use of digital media and social platforms that may delay sleep onset. By concentrating on recent literature, the present study aims to better capture the current relationship between sleep quality and academic performance as experienced by today’s children. Nevertheless, future meta-analyses could extend the time frame to examine how these relationships may have evolved over longer historical periods.

### Implications

The present findings indicate that sleep quality is a meaningful, though modest, correlate of academic performance across childhood and adolescence, particularly during the elementary and adolescent years. These results suggest that sleep may be considered one relevant contextual factor influencing students’ readiness to learn, attention, and classroom engagement. However, sleep represents only one of many interacting influences on academic outcomes, alongside instructional quality, socioeconomic factors, emotional well-being, and family environment. While improvements in sleep quality alone are unlikely to produce large academic gains, school practices that aim to reduce chronic sleep restriction—such as monitoring academic workload, discouraging late-night academic demands, or considering developmentally appropriate school start times—may contribute to academic functioning when implemented as part of broader educational and psychosocial supports.

Importantly, sleep difficulties may also reflect broader challenges, including emotional distress or family stress, rather than being interpreted solely as issues of motivation or effort. Although the present findings are correlational and do not establish causality, they complement experimental research by identifying the magnitude and developmental patterning of sleep–academic associations under naturalistic conditions. Future intervention studies are needed to determine whether changes in sleep lead to corresponding improvements in academic functioning.

Overall, the results underscore that sufficient and high-quality sleep is one potentially modifiable factor that may support children’s learning and cognitive development, while acknowledging that academic success is shaped by multiple, interacting influences.

## Figures and Tables

**Table 1:** Included studies for meta-analysis.

Author(s) and Year	N	Age	Sleep Assessment	Achievement Assessment	Cultural Background	Inclusion of Variables
(Chen, 2018) *	254	13–18	Actigraphy	School transcript	E ^1^	SQ
(Fang, 2021) *	175	7–12	Report-by-others	Report-by-others	E ^1^	SQ SD SJ SL DD SDI
(Fang, 2021) *	203	7–12	Report-by-others	School transcript	E ^1^	SQ SD DD SDI
(Fang et al., 2023)	203	7–12	Report-by-others	School transcript	E ^1^	SQ
(Lin & Yi, 2014)	2483	13–18	Self-report	Self-report	E ^1^	SQ SD SJ
(J. Liu et al., 2019)	185	7–12	Self-report	School transcript	E ^1^	SQ SD
(J. Liu et al., 2019)	201	7–12	Self-report	School transcript	E ^1^	SQ SD
(J. Liu et al., 2019)	218	7–12	Self-report	School transcript	E ^1^	SQ SD
(Zhao, 2022) *	534	7–12	Self-report	School transcript	E ^1^	SQ SD SDI DD BT
(Sivakumar et al., 2022)	791	7–12	Report-by-others	School transcript	E ^2^	SQ SDI
(Singh et al., 2018)	501	other	Self-report	School transcript	E ^2^	SQ SD
(Guclu et al., 2024)	1959	13–18	Self-report	School transcript	E ^3^	SQ
(Masal et al., 2016)	3677	other	Self-report	Self-report	E ^3^	SQ SD SJ
(Anjarwala et al., 2020)	440	other	Self-report	School transcript	E ^4^	SQ SD
(Shimura et al., 2022)	294	13–18	Other	Self-report	E ^5^	SQ DD
(Kolomeichuk et al., 2016)	1666	other	Self-report	Self-report	W ^1^	SQ SD
(Stefansdottir et al., 2022)	253	13–18	Actigraphy	School transcript	W ^2^	SQ SJ
(Lien et al., 2020)	3196	7–12	Self-report	Self-report	W ^3^	SQ SD
(Lien et al., 2020)	6964	other	Self-report	Self-report	W ^3^	SQ SD
(Lien et al., 2020)	10,160	other	Self-report	Self-report	W ^3^	SQ SD
(Dubuc et al., 2019)	187	13–18	Self-report	School transcript	W ^3^	SQ SD
(Faught et al., 2017b)	28,608	other	Self-report	Self-report	W ^3^	SQ SD
(Faught et al., 2017a)	4253	7–12	Self-report	School transcript	W ^3^	SQ SD
(Faught et al., 2019)	11,016	13–18	Self-report	School transcript	W ^3^	SQ SD
(Arrona-Palacios & Díaz-Morales, 2018)	400	13–18	Self-report	School transcript	W ^4^	SQ SJ SD
(Gruber et al., 2014)	70	7–12	Actigraphy	School transcript	W ^4^	SQ
(Alexopoulou et al., 2024)	831	13–18	Self-report	School transcript	W ^5^	SQ SD
(Perkinson-Gloor et al., 2013)	2716	13–18	Self-report	School transcript	W ^6^	SQ SD
(Tonetti et al., 2015)	37	13–18	Actigraphy	School transcript	W ^7^	SQ SD
(Reale et al., 2013)	154	7–12	Report-by-others	Report-by-others	W ^7^	SQ SDI
(Hysing et al., 2016)	7798	13–18	Self-report	School transcript	W ^8^	SQ SJ SD BT
(Husarova et al., 2018)	7595	other	Self-report	School transcript	W ^9^	SQ SL SD
(Maniraj et al., 2023)	322	7–12	Self-report	School transcript	W ^10^	SQ
(Howie et al., 2020)	934	other	Self-report	Other	W ^11^	SQ SD
(Howie et al., 2020)	934	other	Self-report	School transcript	W ^11^	SQ SD
(Orihuela et al., 2023)	288	7–12	Other	School transcript	W ^12^	SQ SD DD
(Lewin et al., 2017)	26,440	7–12	Self-report	Self-report	W ^12^	SQ SD
(Teti et al., 2022)	221	0–6	Actigraphy	Report-by-others	W ^12^	SQ SD
(Fuligni et al., 2018)	341	13–18	Self-report	School transcript	W ^12^	SQ SD
(Hoyniak et al., 2020)	119	0–6	Actigraphy	Report-by-others	W ^12^	SQ SL SJ SD BT
(Asarnow et al., 2014)	2700	13–18	Self-report	School transcript	W ^12^	SQ SD
(Wernette & Emory, 2017)	217	13–18	Self-report	School transcript	W ^12^	SQ SD
(El-Sheikh et al., 2019)	282	7–12	Actigraphy	Report-by-others	W ^12^	SQ SD
(El-Sheikh et al., 2019)	282	7–12	Actigraphy	School transcript	W ^12^	SQ SD
(Stroebele et al., 2013)	1057	7–12	Self-report	Self-report	W ^12^	SQ SD
(Patel et al., 2020)	11	7–12	Actigraphy	School transcript	W ^12^	SQ SD
(Patel et al., 2020)	17	7–12	Actigraphy	School transcript	W ^12^	SQ SD DD
(Pecor et al., 2016)	976	13–18	Self-report	School transcript	W ^12^	SQ BT SD
(Rea-Sandin et al., 2022)	707	7–12	Self-report	Report-by-others	W ^12^	SQ SD SDI
(Berger et al., 2018)	103	0–6	Actigraphy	Report-by-others	W ^12^	SQ SD
(Rubens et al., 2020)	105	7–12	Self-report	School transcript	W ^12^	SQ DD
(Burns et al., 2018)	4625	13–18	Self-report	Self-report	W ^12^	SQ SD
(Short et al., 2013)	385	13–18	Self-report	Self-report	W ^12^	SQ SD
(Erath et al., 2015)	280	7–12	Actigraphy	School transcript	W ^12^	SQ
(Adelantado-Renau et al., 2019a)	257	13–18	Self-report	School transcript	W ^13^	SQ SD DD SDI
(Adelantado-Renau et al., 2019a)	257	13–18	Actigraphy	School transcript	W ^13^	SQ SD DD SDI
(Adelantado-Renau et al., 2019b)	269	13–18	Self-report	School transcript	W ^13^	SQ SD
(Adelantado-Renau et al., 2019b)	269	13–18	Other	School transcript	W ^13^	SQ SD
(Diaz et al., 2017)	103	0–6	Actigraphy	Report-by-others	W ^13^	SQ SL BT
(Peiró-Velert et al., 2014)	3095	other	Self-report	School transcript	W ^13^	SQ SD
(Torres-Lopez et al., 2022)	85	7–12	Self-report	School transcript	W ^13^	SQ SDI
(Torres-Lopez et al., 2022)	109	7–12	Self-report	Report-by-others	W ^13^	SQ DDI
(Tapia-Serrano et al., 2022a)	1277	other	Self-report	School transcript	W ^13^	SQ SD
(Tapia-Serrano et al., 2022b)	844	13–18	Self-report	School transcript	W ^13^	SQ SD
(Merayo et al., 2022)	261	13–18	Report-by-others	School transcript	W ^13^	SQ SD
(Perez-Lloret et al., 2013)	1194	13–18	Report-by-others	School transcript	W ^14^	SQ SD DD

Note. Authors and year with “*” indicates Thesis; N = sample size; W = Western culture (W: ^1^ = Russia, ^2^ = Iceland, ^3^ = Canada, ^4^ = Mexico, ^5^ = Greece, ^6^ = Germany, ^7^ = Italy, ^8^ = Norway, ^9^ = Slovakia, ^10^ = France, ^11^ = Australia, ^12^ = United States, ^13^ = Spain, ^14^ = Argentina; E = Eastern culture (E: ^1^ = China, ^2^ = India, ^3^ = Turkey, ^4^ = Pakistan, ^5^ = Japan); Actigraphy = including accelerometers, electronic watches, etc.; Self-report = including verbal report, questionnaires, scale, etc.; Other = including a variety of mixed methods, weighted forms, etc.; SQ = Sleep Quality; SD = Sleep Duration; SDI = Sleep Disorders; SL = Sleep Latency; SJ = Social Jetlag; BT = Bedtime; DD = Daytime Dysfunction.

**Table 2:** Summary of meta-analytic results across moderators.

Measure		k	Effect Size (r)	df	95% CI	τ^2^
Main Average Effect Size						
	Sleep Quality	72.0	0.017	71.0	[0.139, 0.200]	0.015
Sleep Components						
	Bedtime	8.0	−0.240	7.0	[−0.457, 0.004]	0.123
	Social jetlag	11.0	−0.104	10.0	[−0.138, −0.070]	0.008
	Sleep Latency	8.0	−0.154	7.0	[−0.214, −0.092]	0.003
	Sleep Duration	55.0	0.132	54.0	[0.099, 0.164]	0.012
	Daytime Dysfunction	10.0	−0.238	9.0	[−0.394, −0.007]	0.07
School Subjects						
	Mathematics	16.0	0.206	15.0	[0.109, 0.298]	0.046
	Languages	16.0	0.213	15.0	[0.116, 0.305]	0.031
	Science	4.0	0.020	3.0	[−0.176, 0.215]	0.0
Age						
	Age 0–6 (pre-school)	7.0	0.096	6.0	[−0.013, 0.157]	0.005
	Age 7–12 (elementary school)	25.0	0.230	24.0	[0.161, 0.296]	0.027
	Age 13–18 (secondary school)	28.0	0.139	28.0	[0.093, 0.185]	0.014
Culture						
	Eastern culture	15.0	0.284	14.0	[0.154, 0.404]	0.069
	Western culture	57.0	0.138	56.0	[0.108, 0.168]	0.011
Research Methods—Sleep Quality						
	Self-reported	45.0	0.16	44.0	[0.123, 0.195]	0.014
	Actigraphy	17.0	0.127	16.0	[0.044, 0.209]	0.021
	Reported by others	7.0	0.284	6.0	[0.139, 0.417]	0.038
Research Methods—Academic Performance						
	Self-reported	14.0	0.127	13.0	[0.084, 0.170]	0.006
	School report cards	46.0	0.193	45.0	[0.139, 0.245]	0.032
	Reported by others	11.0	0.121	10.0	[0.043, 0.197]	0.011

*Note*. r = correlation coefficient; k = number of effect sizes; τ^2^ = between-study variance. Random-effects model used.

## Data Availability

The original contributions presented in this study are included in the article/Supplementary Materials. Further enquiries can be directed to the corresponding author.

## Note

1 References (Adelantado-Renau et al., 2019a, 2019b; Alexopoulou et al., 2024; Anjarwala et al., 2020; Arrona-Palacios & Díaz-Morales, 2018; Asarnow et al., 2014; Berger et al., 2018; Burns et al., 2018; Cole, 2016; Cooper et al., 2012; Diaz et al., 2017; Díaz-Morales et al., 2015; Dubuc et al., 2019; El-Sheikh et al., 2019; Erath et al., 2015; Fang, 2021; Faught et al., 2017a, 2017b, 2019; Fuligni et al., 2018; Gruber et al., 2016, 2014; Guclu et al., 2024; Howie et al., 2020; Hoyniak et al., 2020; Husarova et al., 2018; Hysing et al., 2016; Kolomeichuk et al., 2016; Lewin et al., 2017; Lien et al., 2020; Lin & Yi, 2014; J. Liu et al., 2019; Y. Liu et al., 2022; Maniraj et al., 2023; Masal et al., 2016; Merayo et al., 2022; Orihuela et al., 2023; Patel et al., 2020; Pecor et al., 2016; Peiró-Velert et al., 2014; Perez-Lloret et al., 2013; Perkinson-Gloor et al., 2013; Reale et al., 2013; Rea-Sandin et al., 2022; Rubens et al., 2020; Sánchez-Hernando et al., 2021; Shimura et al., 2022; Short et al., 2013; Singh et al., 2018; Sivakumar et al., 2022; Stefansdottir et al., 2022; Stroebele et al., 2013; Tapia-Serrano et al., 2022a, 2022b; Teti et al., 2022; Tonetti et al., 2015, 2016; Torres-Lopez et al., 2022; Wernette & Emory, 2017; Zhao, 2022) are used for meta analysis.

## Supplementary Materials

The following supporting information can be downloaded at: https://www.mdpi.com/article/10.3390/bs16050634/s1, The PRISMA checklist.
