# Supplementary material for: The Effect of Sleep Quality on Academic Performance: A Systematic Review and Meta-Analysis Study
Source: Behav Sci (Basel). 2026 Apr 23;16(5):634. doi: 10.3390/bs16050634 (PMC13203443; doi:10.3390/bs16050634)
Supplement: Supplementary file 1 [file behavsci-16-00634-s001.zip › behavsci-4145638-supplementary.pdf]

PRISMA 2020 Checklist

This PRISMA 2020 checklist has been completed for the manuscript titled "The Effect of Sleep Quality on Academic Performance: A Meta-Analysis Study." Page numbers correspond to the uploaded manuscript version (V6).

Section/Topic	Checklist Item	Location in Manuscript
Title	Identify the report as a systematic review.	Page 1 (Title)
Abstract	Structured summary of review.	Page 1 (Abstract)
Rationale	Describe the rationale for the review.	Page 2 (Introduction)
Objectives	Provide an explicit statement of the objective(s).	Page 4 (PICO)
Eligibility criteria	Specify inclusion and exclusion criteria.	Page 6 (Eligibility Criteria section)
Information sources	Describe all information sources.	Page 5 (Information Source and Search Strategy)
Search strategy	Present full search strategy.	Page 5 (Information Source and Search Strategy)
Selection process	State the process for selecting studies.	Page 6 (Eligibility Criteria, Fig 1)
Data collection process	Specify methods of data collection.	Page5 (Coding section)
Data items	List and define all variables for which data were sought.	Page 10 (Coding, Table 1)
Study risk of bias assessment	Describe any methods used to assess risk of bias.	Page 6 (Risk-of-Bias Assessment)
Effect measures	Specify effect measures used.	Page 7 (Calculation and Analysis of Effect Sizes)
Synthesis methods	Describe methods for synthesis.	Page 7 (Data Analysis)

Reporting bias assessment	Describe methods to assess risk of bias due to missing results.	Page 7 (Risk-of-Bias Results)
Certainty assessment	Describe any methods used to assess certainty in the body of evidence.	Page 8 (Heterogeneity testing)
Study selection	Give numbers of studies screened, assessed and included, with reasons.	Page 5 (Eligibility Criteria, Fig 1)
Study characteristics	Present characteristics of each study.	Page 10 (Table 1)
Risk of bias in studies	Present risk of bias assessments for each study.	Page 8 (Publication Bias section, Fig 2)
Results of individual studies	For all outcomes, present summary data for each study.	Page 9 (Fig. 3)
Results of syntheses	Summarize characteristics and results of syntheses.	Page 8-12 (Results section)
Reporting biases	Present results of assessments of risk of bias due to missing results.	Page 8 (Heterogeneity Testing)
Certainty of evidence	Present assessments of certainty of evidence.	Page 8 (Heterogeneity Testing)
Discussion	Summarize main findings, limitations, conclusions.	Page 13–18 (Discussion, Limitations, Implication)
Registration	Provide registration information.	Not registered
Protocol	Indicate if a protocol exists.	Not reported
Support	Describe sources of support, role of funders.	Page 16 (Funding)
Competing interests	Declare competing interests.	Page 16 (Conflicts of Interest)
